# Therapeutic Potentials of Marine-Derived Compounds in Rheumatoid Arthritis

**DOI:** 10.3390/nu18152558

**Published:** 2026-08-05

**Authors:** Rowena Thekkekara, Anupama Bangra Kulur, Jamie Seymour, Haleagrahara Nagaraja

**Affiliations:** 1 College of Medicine and Dentistry, James Cook University, Townsville, QLD 4811, Australia; 2 Australian Institute of Tropical Health and Medicine (AITHM), James Cook University, Cairns, QLD 4870, Australia

**Keywords:** rheumatoid arthritis, marine natural products, anti-inflammatory, immunomodulation, marine microalgae, omega-3 fatty acids, astaxanthin, fucoxanthin, NF-κB, cytokines

## Abstract

Rheumatoid arthritis (RA) is a chronic, systemic autoimmune disease defined by persistent synovial inflammation, progressive cartilage and bone erosion, and extra-articular manifestations. Current treatments, such as disease-modifying anti-rheumatic drugs (DMARDs), glucocorticoids, and non-steroidal anti-inflammatory drugs (NSAIDs), control disease activity but are limited by toxicity, reduced response over time, and are expensive, demonstrating the need for safer and more effective adjuncts. The marine environment is a rich, largely untapped source of structurally diverse bioactive molecules that could be used to develop new therapeutics. This review compiles current evidence on anti-inflammatory and immunomodulatory compounds from marine organisms relevant to RA, including macroalgae, true marine microalgae, marine microorganisms (bacteria and fungi, including deep-sea taxa), sea cucumbers, sponges, mussels, corals, and jellyfish. Recurring mechanisms of action include inhibition of the NF-κB, MAPK, and JAK/STAT signalling cascades; suppression of inducible nitric oxide synthase (iNOS) and cyclooxygenase-2 (COX-2); reduced production of tumour necrosis factor-α (TNF-α), interleukin (IL)-1β, and IL-6; and activation of the Nrf2 antioxidant response. Particular attention is given to functional lipids (eicosapentaenoic and docosahexaenoic acids, prostaglandin-like oxylipins) and pigments (astaxanthin, fucoxanthin, β-carotene) from marine microalgae and heterotrophic protists, which recent literature identifies as the most clinically advanced marine leads. To clarify translational status, compounds are grouped by their development stage (marketed nutraceutical, clinical trial, or preclinical) and summarised in a dedicated table. Some compounds, such as green-lipped mussel extract, microalgal omega-3 oils, and astaxanthin, have reached the stage of randomised controlled trials for arthritis. However, most other potential treatments are still in the early, preclinical phase. It is worth noting that ocean-derived compounds appear generally safe, but more thorough studies, especially in living organisms and in clinical settings, are needed to confirm their effectiveness for rheumatoid arthritis before any claims can be made about their therapeutic benefits.

## 1. Introduction

Rheumatoid arthritis (RA) is a chronic, systemic, symmetrical autoimmune polyarthritis that mainly affects small peripheral joints and results in both articular and extra-articular manifestations. It is the most prevalent form of inflammatory arthritis, affecting approximately 0.5–1% of adults globally, and occurs two to three times more frequently in women than in men [1,2,3,4]. Patients often experience joint pain, swelling, and stiffness, especially in the morning. If the condition worsens, it can cause joint deformities, including ulnar deviation, boutonniere deformity, and swan-neck deformity [1]. Modern RA management, based on early diagnosis and treat-to-target strategies incorporating methotrexate, biologic DMARDs, and targeted synthetic agents, has substantially enhanced outcomes, achieving remission or low disease activity in many patients [2,5]. A substantial proportion of patients do not achieve sustained remission, lose response over time, or experience treatment-limiting adverse effects or high treatment costs, sustaining interest in complementary, naturally derived anti-inflammatory agents as potential adjuncts rather than replacements [2,6]. The ocean, covering more than 70% of the Earth’s surface, harbors extraordinary phylogenetic and chemical diversity; marine organisms biosynthesise structurally unique secondary metabolites as chemical defenses, many of which interfere with the inflammatory pathways connected in RA [7]. This review evaluates the anti-inflammatory and immunomodulatory potential of marine-derived compounds relevant to RA, focusing on organisms such as macroalgae, marine microalgae and heterotrophic protists, marine bacteria and fungi, sea cucumbers, sponges, mussels, corals, and jellyfish. For each group, the active compounds, their molecular targets, and the level of experimental evidence are summarised, and the progression of these leads toward clinical application is appraised.

### Scope and Search Strategy

This article is a narrative review. The relevant literature was identified by searching PubMed, Scopus and Web of Science for articles published up to June 2025, using combinations of the terms ‘marine’, ‘algae’, ‘microalgae’, ‘sponge’, ‘coral’, ‘mussel’, ‘sea cucumber’, ‘jellyfish’, ‘bacteria’, ‘fungi’, ‘natural product’, ‘anti-inflammatory’, ‘immunomodulatory’, ‘rheumatoid arthritis’ and ‘inflammation’, supplemented by hand-searching of the reference lists of retrieved articles and relevant reviews. Studies were selected based on relevance to the anti-inflammatory or immunomodulatory activity of marine-derived compounds and their potential relevance to RA; in vitro and in vivo mechanistic studies and human trials were considered, with priority given to original research and more recent work (predominantly 2010–2025). Consistent with a narrative design, no formal quantitative screening, risk-of-bias assessment or meta-analysis was undertaken, and the selection is intended to be representative rather than exhaustive.

## 2. Pathophysiology

**Figure 1:**
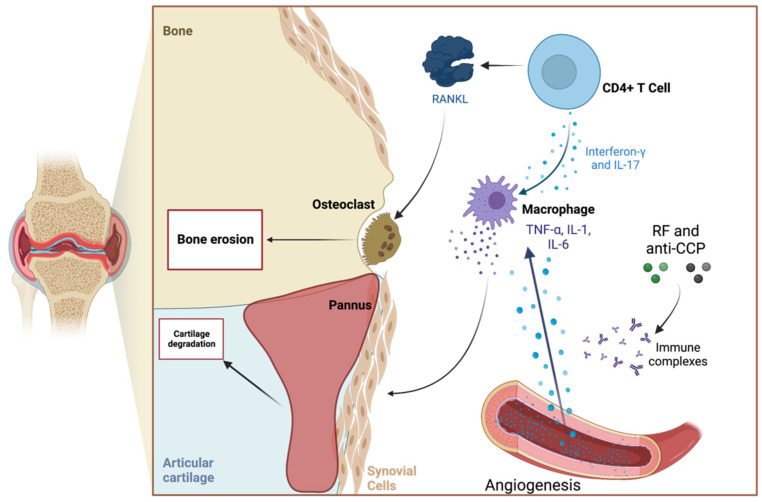
Pathophysiology of rheumatoid arthritis (created with BioRender.com).

RA evolves through a pre-clinical phase in which immune dysregulation precedes overt synovitis. During this period, loss of immune tolerance to post-translationally modified self-proteins drives the generation of autoantibodies, notably rheumatoid factor (RF) and anti-citrullinated protein antibodies (ACPAs), in genetically predisposed individuals exposed to environmental triggers [8]. Susceptibility is strongly associated with the HLA-DRB1 ’shared epitope’ alleles (e.g., HLA-DR1 and HLA-DR4), while smoking, periodontal pathogens and mucosal dysbiosis can initiate citrullination and break tolerance [8,9]. Clinically apparent disease is established once chronic synovial inflammation persists for at least six weeks [8]. In the established phase, activated CD4^+^ T cells secrete interferon-γ (IFN-γ) and IL-17, recruiting and activating macrophages that release the pivotal pro-inflammatory cytokines TNF-α, IL-1β and IL-6 [9]. These cytokines drive proliferation and activation of fibroblast-like synoviocytes (FLS), which are now recognised as central, semi-autonomous effectors of joint degradation: activated FLS resist apoptosis, secrete matrix metalloproteinases (MMPs) and chemokines, and form an invasive, hyperplastic pannus that erodes cartilage and bone [9,10]. Pro-inflammatory cytokines also upregulate receptor activator of nuclear factor κB ligand (RANKL), which binds to RANK on osteoclast precursors to promote osteoclastogenesis and bone erosion, a process partially counterbalanced by anti-inflammatory mediators such as IL-10 [9]. Immune complexes formed by RF and ACPAs activate the complement system and amplify synovitis, while sustained angiogenesis supports the supply of inflammatory cells [9]. Systemic spread of these mediators underlies extra-articular features, including rheumatoid nodules, anaemia, pleuropericardial disease, accelerated atherosclerosis and constitutional symptoms such as fatigue, fever and weight loss [6,9]. The principal intracellular signalling hubs in this network, NF-κB, mitogen-activated protein kinases (MAPKs) and the JAK/STAT axis, are the very pathways most frequently targeted by the marine-derived compounds reviewed below (Figure 1).

## 3. Conventional Pharmacological Therapies and Their Limitations

### 3.1. Disease-Modifying Anti-Rheumatic Drugs (DMARDs)

**Figure 2:**
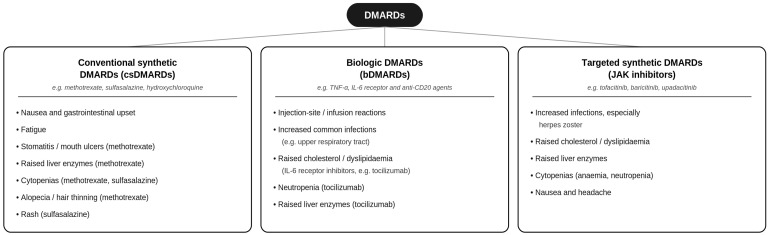
Common adverse effects of disease-modifying anti-rheumatic drugs (DMARDs), grouped by class. Only adverse effects that are common are shown; effects that are characteristic of specific agents rather than of the whole class are indicated in parentheses. csDMARDs, conventional synthetic DMARDs; bDMARDs, biologic DMARDs; IL-6, interleukin-6; JAK, Janus kinase; TNF-α, tumour necrosis factor-α.

Although rheumatoid arthritis cannot be cured, controlling inflammation early and consistently can help prevent or slow joint damage. DMARDs are the basis of treatment and are most effective when started early; they are slow-acting, with a delayed onset of approximately 6 weeks to 6 months [5]. DMARDs are grouped into conventional synthetic (csDMARDs), biologic (bDMARDs) and targeted synthetic (tsDMARDs) classes, generally introduced in that sequence [5,11]. Methotrexate, a csDMARD, remains the anchor first-line agent because of its benefit, low cost and well-characterised safety profile; it is frequently combined with short-term glucocorticoids at diagnosis to achieve rapid control, and 30–50% of patients with early RA attain low disease activity or remission on methotrexate-based regimens [12,13,14,15,16]. Methotrexate additionally improves the efficacy of tsDMARDs and bDMARDs when co-administered [13,17]. Other conventional synthetic agents used alongside or instead of methotrexate include hydroxychloroquine and sulfasalazine. When csDMARD therapy is insufficient, biologic DMARDs, such as tumour necrosis factor-α (TNF-α) inhibitors (e.g., infliximab), interleukin-6 (IL-6) receptor inhibitors, B-cell-depleting (anti-CD20) antibodies and T-cell co-stimulation blockers, or targeted synthetic Janus kinase (JAK) inhibitors, are added, most often in combination with methotrexate [5]. Despite their effectiveness, DMARDs carry major drawbacks (Figure 2). The adverse-effect profile is agent-specific. Methotrexate commonly causes cytopenias, hepatotoxicity, stomatitis and gastrointestinal intolerance, with rare pneumonitis; folate-antagonism toxicity (stomatitis, alopecia, nausea) is mitigated by folate supplementation [5,18]. Other csDMARDs may cause retinopathy (hydroxychloroquine) or cytopenias and rash (sulfasalazine). Among biologics, TNF-α inhibitors increase the risk of serious and opportunistic infections and of reactivation of latent tuberculosis, and may unmask demyelinating disease or worsen congestive heart failure, whereas IL-6 receptor inhibitors (e.g., tocilizumab) frequently cause dyslipidaemia, neutropenia and transaminase elevation [19,20]. Targeted synthetic JAK inhibitors carry class risks of herpes zoster, venous thromboembolism and dyslipidaemia [5]. The need to cycle through agents to identify an effective, tolerated regimen adds considerable time and cost to care [21].

### 3.2. Glucocorticoids

Glucocorticoids such as prednisolone produce rapid, broad anti-inflammatory effects, largely through transrepression of pro-inflammatory gene expression, and can slow early radiographic progression [22]. However, glucocorticoids are not recommended as part of routine long-term RA management and are best reserved for short-term, bridging use to obtain rapid control of inflammatory symptoms or disease flares, because long-term exposure causes multisystem metabolic and skeletal toxicity, including osteoporosis, hyperglycaemia, gastrointestinal ulceration and bleeding, hypertension and increased infection risk [22].

### 3.3. Non-Steroidal Anti-Inflammatory Drugs (NSAIDs)

NSAIDs such as aspirin, diclofenac, flurbiprofen and ibuprofen relieve pain and stiffness and improve joint function, but they are symptomatic agents only and do not prevent structural joint damage [23]. Their anti-inflammatory action derives from inhibition of cyclooxygenase-mediated prostanoid biosynthesis, decreasing prostaglandin E2, thromboxane A2, prostacyclin and related mediators that signal through G-protein-coupled receptors [24]. Because these prostanoids also serve homeostatic roles, NSAID therapy carries a well-recognised risk of gastrointestinal, renal, hepatic and cardiovascular adverse effects [23,25]. The cumulative toxicity of DMARDs, glucocorticoids and NSAIDs provides the central rationale for seeking better-tolerated, naturally derived anti-inflammatory adjuncts.

## 4. Marine-Derived Compounds with Anti-Inflammatory Activity

**Figure 3:**
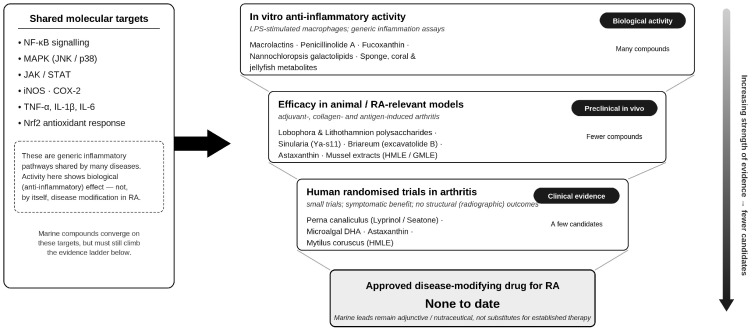
Translational overview of marine-derived anti-inflammatory compounds in rheumatoid arthritis (RA). Representative compounds are mapped from the shared molecular targets they modulate (left) through the level of experimental evidence to their translational stage (funnel): in vitro activity, efficacy in animal/RA-relevant models, human randomised trials, and approved disease-modifying drugs. Colour denotes stage; no marine-derived compound is yet approved as a disease-modifying agent for RA.

Marine organisms are a prolific source of anti-inflammatory chemistry, with over 1500 new marine natural products described each year [7]. Compounds investigated as potential RA-relevant therapies act by inhibiting one or more inflammatory mediators, including TNF-α, IL-1β, IL-6, IL-17, IFN-γ, nitric oxide (NO), and thromboxanes, as well as the upstream enzymes iNOS and COX-2 [7,26]. The sections that follow are organised by source organism: macroalgae, marine microalgae and heterotrophic protists, marine microorganisms (bacteria and fungi), sea cucumbers, sponges, mussels, corals and jellyfish. It should be noted that much of the primary literature studies these compounds in generic models of inflammation (typically lipopolysaccharide [LPS]-stimulated macrophages or carrageenan/zymosan-induced inflammation) rather than in validated RA models; the relevance of those findings to RA is therefore inferred from shared mechanisms and is made explicit throughout (Figure 3). NF-κB, iNOS, COX-2, and pro-inflammatory cytokine production are generic inflammatory mechanisms common to many diseases. Their inhibition in vitro or in non-RA models demonstrates biological anti-inflammatory activity but does not, by itself, establish disease-modifying potential in RA. This distinction between mechanistic plausibility and clinically meaningful therapeutic effect is maintained throughout the review.

### 4.1. Macroalgae

#### 4.1.1. *Lobophora variegata*

The brown alga *Lobophora variegata* has been studied in a rat model of zymosan-induced arthritis [27]. Heterofucans, sulphated polysaccharides composed of fucose and uronic acids, exhibited both anti-inflammatory and antioxidant activity, modulating the cellular redox state. The acetone-derived fraction F1 scavenged reactive oxygen species (superoxide and hydroxyl radicals) and, administered intraperitoneally at 25–75 mg/kg, reduced leukocyte influx by 52.1–96.7% and NO levels by 27.2–39% relative to untreated arthritic controls; reductions in paw oedema and serum TNF-α were significant at 50 mg/kg (*p* < 0.001) [27]. Immunohistochemistry confirmed reduced synovial cell infiltration and TNF-α expression. The authors attributed these effects to redox modulation by the fucosylated, sulphated saccharides, although high polysaccharide viscosity may have inflated some in vitro antioxidant readouts [27]. Overall, *L. variegata* heterofucans display antioxidant-linked anti-inflammatory activity in an established arthritis model.

#### 4.1.2. *Lithothamnion muelleri*

The calcareous red alga *Lithothamnion muelleri* attenuated inflammation and hypernociception in the murine antigen-induced (mBSA) arthritis model [28]. Treatment reduced inflammation in a time- and dose-dependent manner, decreasing the number and activation of leukocytes in draining lymph nodes and the knee joint and lowering the neutrophil chemokines CXCL1 and CXCL2. Intravital microscopy showed that the alga, or its polysaccharide-rich fraction, suppressed firm leukocyte adhesion to the endothelium, thereby limiting neutrophil recruitment, tissue damage and hypernociception; notably, joint IFN-γ and TNF-α were not significantly altered, showing a predominantly chemokine- and adhesion-dependent mechanism [28]. *L. muelleri* also reduced activated CD4^+^ CD25^+^ T cells and CD11c^+^ CD86^+^ dendritic cells in draining popliteal lymph nodes without impairing immunisation, pointing to immunomodulatory activity of its sulphated polysaccharides [28]. These findings show effective control of the articular inflammatory response in a recognised model of arthritis.

### 4.2. Marine Microalgae and Heterotrophic Protists

Marine microalgae are a diverse group of photosynthetic protists that accumulate two principal classes of anti-inflammatory metabolites: long-chain polyunsaturated fatty acids (PUFAs) and pigments (carotenoids). Their relevance to RA was recently reviewed in detail by Cutolo and colleagues [29], who emphasised that several genuinely marine taxa, together with related heterotrophic protists, are among the most promising and clinically advanced marine leads. We note that Chlorella vulgaris, which has frequently been cited in this context, is in fact a freshwater chlorophyte rather than a marine species; although its extracts inhibit TNF-α, IL-6 and NO production in LPS-stimulated RAW 264.7 macrophages in a dose-dependent manner [30], it is included here only for comparison, and the emphasis below is placed on true marine producers.

#### 4.2.1. Functional Lipids (EPA, DHA and Prostaglandin-like Oxylipins)

Many marine microalgae are rich in the omega-3 PUFAs eicosapentaenoic acid (EPA, 20:5 *n*-3) and docosahexaenoic acid (DHA, 22:6 *n*-3), which are precursors of specialised pro-resolving lipid mediators and which suppress NF-κB signalling [29]. The eustigmatophyte *Nannochloropsis* is an established marine EPA producer; organic-solvent extracts of *Nannochloropsis oculata* inhibited NO production in LPS-stimulated RAW 264.7 macrophages in a dose-dependent manner [31], and the galactolipids monogalactosyl- and digalactosyldiacylglycerol (MGDG and DGDG) isolated from *Nannochloropsis granulata* downregulated iNOS protein and inhibited NO production without cytotoxicity [32]. Lipid extracts from the EPA/DHA-rich haptophyte *Pavlova lutheri* and from *Nannochloropsis* spp. reduced IL-6 and TNF-α secretion by activated human macrophages, consistent with NF-κB pathway suppression [33]. Heterotrophic protists are particularly attractive industrial sources of omega-3 oils: thraustochytrids of the genera *Schizochytrium* and *Aurantiochytrium* are strong DHA producers, can be fermented on renewable feedstocks and have obtained Novel Food status [29]. Importantly, a randomised, double-blind, placebo-controlled crossover trial showed that an EPA/DHA-enriched oil from *Schizochytrium* sp. (2.1 g DHA/day for 10 weeks) significantly reduced the combined count of tender and swollen joints in patients with RA [34], providing direct clinical support for microalgal omega-3 oils as RA adjuncts. Several diatoms (e.g., *Skeletonema marinoi*, *Thalassiosira rotula*) and the haptophyte *Tisochrysis lutea* additionally synthesise prostaglandin-like oxylipins and isoprostanoids that modulate inflammation and may protect bone by inhibiting osteoclast differentiation [29].

#### 4.2.2. Pigments (Astaxanthin, Fucoxanthin and β-Carotene)

Microalgal carotenoids are potent antioxidants that interfere with the major pro-inflammatory signalling pathways connected in RA [29]. Astaxanthin, a ketocarotenoid produced commercially from the alga *Haematococcus* and accumulated by some marine protists, is an exceptionally efficient scavenger of reactive oxygen species; it suppresses NF-κB and JNK/p38 MAPK signalling, activates the Nrf2 antioxidant response, inhibits RANKL-induced osteoclastogenic gene expression in macrophages, and promotes cartilage health in animal models of arthritis and osteoarthritis [29]. These preclinical observations are now supported by clinical evidence: in a randomised, triple-blind, placebo-controlled trial in 60 patients with RA, astaxanthin (20 mg/day for 8 weeks) significantly reduced the Disease Activity Score-28 (DAS-28), Health Assessment Questionnaire (HAQ) score and erythrocyte sedimentation rate compared with placebo [35]. Fucoxanthin, a xanthophyll abundant in marine diatoms (e.g., *Phaeodactylum tricornutum*) and the haptophyte *Tisochrysis lutea*, inhibits the inflammatory response by suppressing NF-κB and MAPK activation in LPS-stimulated RAW 264.7 macrophages, reducing NO, prostaglandin E2, iNOS, COX-2, TNF-α, IL-1β and IL-6 [36]. The carotene β-carotene, produced in abundance by the marine halophile *Dunaliella salina*, and the xanthophyll lutein exert additional anti-inflammatory effects through NF-κB, JNK/p38 MAPK and JAK2/STAT3 inhibition [29]. Collectively, marine microalgal pigments and lipids constitute the most mechanistically and clinically substantiated group of marine leads for RA.

### 4.3. Marine Microorganisms (Bacteria and Fungi)

Marine bacteria and fungi, including strains from extreme deep-sea and symbiotic niches, are an exceptionally productive but, in the RA context, previously overlooked source of anti-inflammatory secondary metabolites. A systematic survey of 2021–2023 catalogued approximately 252 anti-inflammatory compounds from marine microorganisms, of which 129 were new structures, predominantly polyketides, terpenoids and alkaloids [26]. Among marine bacteria, macrolactins are a well-known example: 7,13-epoxyl-macrolactin A, isolated from Bacillus subtilis B5 obtained from sediment at a depth of approximately 3000 m, significantly inhibited the mRNA expression of iNOS, IL-1β and IL-6 in LPS-stimulated RAW 264.7 macrophages, with greater potency than previously known macrolactins [37]. Related macrolactins suppress inflammatory mediators by inhibiting Toll-like receptor 4 (TLR4) signalling and inducing the HO-1/Nrf2 antioxidant axis, while cyclic dipeptides from marine Streptomyces spp. inhibit TNF-α and IL-6 production in stimulated macrophages [26]. Marine fungi are equally prolific: a comprehensive survey identified 285 anti-inflammatory metabolites (156 of them novel) reported from marine-derived fungi between 2018 and 2024, sourced from seawater, sediments, mangroves, sponges and corals [26]. A representative primary example is penicillinolide A, a 10-membered lactone from the marine fungus *Penicillium* sp. SF-5292, which inhibited NO and prostaglandin E2 production by downregulating iNOS and COX-2 and reduced TNF-α, IL-1β and IL-6 through suppression of IκB-α phosphorylation and NF-κB nuclear translocation [38]. The convergence of these compounds on the same NF-κB-, TLR4- and Nrf2-dependent mechanisms that direct RA synovitis, combined with the tractability and scalability of microbial fermentation, makes marine microorganisms a promising, though currently entirely preclinical, frontier for future anti-inflammatory drug discovery.

### 4.4. Sea Cucumber

#### *Isostichopus badionotus* 

The sea cucumber *Isostichopus badionotus* has shown anti-inflammatory activity in vivo. Body-wall preparations reduced inflammation in mouse models, an effect attributed to bioactive constituents, such as fucosylated chondroitin sulphates, fucoidans, and lectins [39]. Although the precise mechanism was not fully defined, the activity is thought to involve inhibition of the NF-κB pathway, with reduced COX-2 and TNF expression and impaired cytosolic-to-nuclear translocation of NF-κB, thereby limiting downstream pro-inflammatory gene induction [39].

### 4.5. Sponges

#### 4.5.1. *Hymeniacidon* sp.

Amphilectane diterpenoid metabolites and semi-synthetic analogues from the sponge *Hymeniacidon* sp. inhibit thromboxane B2 (TXB2) generation by activated rat brain microglia through a cyclooxygenase-dependent mechanism, and were more potent than the clinically used NSAIDs aspirin and flurbiprofen [40]. The absence of superoxide inhibition supported a COX-dependent action. These compounds show the potential of sponge metabolites to modulate eicosanoid-driven inflammation, although activity to date is confined to in vitro neuroinflammation models, and their therapeutic window relative to cytotoxicity remains to be defined [40].

#### 4.5.2. *Halichondria sitiens*

Lipophilic fractions from the marine sponge *Halichondria sitiens* decreased secretion of the pro-inflammatory cytokines IL-12p40 and IL-6 by dendritic cells and reduced their capacity to drive a Th1 response in allogeneic CD4^+^ T cells [41]. The sesterterpenoids manoalide and its analogues seco-manoalide and dehydro-seco-manoalide, archetypal sponge-derived phospholipase A2 inhibitors [42], contributed to this activity. These immunomodulatory effects on antigen-presenting cells are mechanistically relevant to RA, but in vivo confirmation is still required [41].

### 4.6. Mussels

#### 4.6.1. *Mytilus coruscus*

A randomised controlled trial evaluated a lipid extract of the hard-shelled mussel *Mytilus coruscus* (HMLE) in 50 patients with RA (aged 28–75 years) who received HMLE capsules or placebo for 6 months [43]. Both the DAS-28 and the Clinical Disease Activity Index improved significantly in the HMLE group (*p* < 0.01), with larger benefits in the per-protocol population. Serum TNF-α, IL-1β and prostaglandin E2 fell in both groups but decreased more markedly with HMLE for TNF-α and prostaglandin E2 (*p* < 0.05), while the anti-inflammatory cytokine IL-10 rose more in the HMLE group (*p* < 0.05) [43].

A complementary preclinical study compared HMLE with New Zealand green-lipped mussel lipid extract (GMLE) in rat models of adjuvant- and collagen-induced arthritis [44]. HMLE at 100 mg/kg showed anti-inflammatory activity comparable to GMLE, reducing hind-paw swelling and the arthritis index and improving body weight gain. The effect was associated with downregulation of inflammatory mediators (LTB4, prostaglandin E2, TXB2), pro-inflammatory cytokines (IL-1β, IL-6, IFN-γ, TNF-α), and MMP-1/MMP-13 expression, together with upregulation of the anti-inflammatory cytokines IL-4 and IL-10, and TIMP-1 in serum and joint tissue [44]. No hepatotoxicity was observed, underscoring the favourable safety profile of mussel lipid extracts.

#### 4.6.2. *Perna canaliculus*

The New Zealand green-lipped mussel, *Perna canaliculus*, is the best-developed marine-derived anti-arthritic nutraceutical. Its lipid fraction contains unstable anti-inflammatory and antioxidant furan fatty acids; after semi-synthetic stabilisation, a furan fatty acid ethyl ester showed anti-inflammatory potency exceeding that of EPA ethyl ester in an adjuvant-induced arthritis model [45]. Stabilisation of the extract (for example, by adding tartaric acid immediately after shucking) yields consistent activity, and the resulting lipid extract is marketed as the dietary supplements Lyprinol and Seatone [45,46]. The traditional use of *P. canaliculus* by Maori communities first prompted this line of investigation [45].

In a double-blind comparison, lipid extracts of *P. canaliculus* (Group A) and green-lipped mussel powder (Group B) both produced considerable improvements in articular index, morning stiffness, and functional index in patients with RA and osteoarthritis [45]. Improvements in grip strength and the visual analogue pain scale were observed but did not reach statistical significance, and benefits were maintained at 6 months. Across the study, the majority of patients improved, with a subset becoming symptom-free [45]. Such trials underpin the standing of *P. canaliculus* lipid extract as a marketed marine nutraceutical for joint inflammation, although effect sizes are modest and findings across formulations have been mixed [47].

### 4.7. Corals

#### 4.7.1. *Briareum excavatum*

The cultured Formosan gorgonian coral *Briareum excavatum* provides several anti-inflammatory diterpenoids. The compound BrD1 inhibited LPS-induced IL-6 and TNF-α expression in mouse bone marrow-derived dendritic cells [48]. The briarane excavatolide B significantly suppressed mRNA expression of the pro-inflammatory enzymes iNOS and COX-2 and, in a carrageenan-induced inflammatory pain model, reduced mechanical allodynia, thermal hyperalgesia, weight-bearing deficits and paw oedema, with diminished iNOS expression and immune-cell infiltration in inflamed tissue [48,49]. These data identify excavatolide B as a marine lead with combined anti-inflammatory and analgesic activity, warranting evaluation in dedicated arthritis models [48,49].

#### 4.7.2. *Sinularia querciformis*

An extract of the soft coral *Sinularia querciformis* showed strong anti-inflammatory activity in mice with experimentally induced arthritis, inhibiting iNOS and COX-2 in macrophages [50]. The active compound, 11-epi-sinulariolide acetate (Ya-s11), reduced inflammatory-cell infiltration into joints and prevented bone destruction, and produced concentration-dependent inhibition of LPS-induced iNOS and COX-2 protein expression in RAW 264.7 macrophages without cytotoxicity, as confirmed by trypan blue and Annexin-V/PI assays [50]. The demonstration of reduced joint inflammation and protection against bone erosion in vivo makes this one of the more RA-relevant coral-derived leads.

#### 4.7.3. *Lemnalia* sp.

A soft coral of the genus *Lemnalia* (family *Nephtheidae*) showed anti-inflammatory activity in a monosodium urate model of gouty arthritis, where intramuscular administration attenuated inflammation and suppressed neutrophil infiltration and related pro-inflammatory cytokines [51]. Additional reported mechanisms include inhibition of Hsp90, induction of apoptosis, and inhibition of COX-2 and IL-8 expression by 11-episinuleptolide acetate via modulation of EGF-mediated cytoplasmic calcium [51].

### 4.8. Jellyfish

Comparatively few studies have explored the anti-inflammatory pharmacology of jellyfish, yet cnidarian peptides display a broad spectrum of antimicrobial, anti-inflammatory and antitumour activities and, given their abundance and high protein content, represent an attractive avenue for discovery [52]. Gold nanoparticles green-synthesised using extracts of the jellyfish *Nemopilema nomurai* (JF-AuNPs) inhibited NO production without cytotoxicity in macrophages and attenuated LPS-induced iNOS gene and protein expression in RAW 264.7 cells in a concentration-dependent manner [53]. Jellyfish venom proteins more broadly possess documented pharmacological potential [54], and a protein extracted from the venom of Pelagia noctiluca inhibited NO production through transcriptional regulation of iNOS in LPS/IFN-γ-stimulated RAW 264.7 macrophages [55]. These early findings show that jellyfish-derived biomolecules merit systematic in vivo investigation.

## 5. Translational Status: From Preclinical Leads to Clinical Use

A recurring criticism of the marine anti-inflammatory literature is that it rarely distinguishes compounds by their stage of development. To address this, we classify the leads discussed above into three categories: (i) products already marketed as dietary supplements or nutraceuticals with supporting clinical data; (ii) compounds evaluated in human clinical trials in arthritis or inflammatory disease; and (iii) compounds at the preclinical (in vitro or animal) stage. This classification is summarised in Table 1.

At present, no marine-derived compound has been approved as a prescription drug specifically for RA. The most clinically mature lead is the green-lipped mussel (*Perna canaliculus*) lipid extract, marketed for decades as Lyprinol and Seatone, which has been evaluated in randomised, follow-up arthritis trials with modest but reproducible symptomatic benefit and a good short-term safety record [45,47]. Marine and microalgal omega-3 (EPA/DHA) oils are widely used adjuncts in RA and are supported by randomised trials, including a controlled study of *Schizochytrium*-derived DHA that reduced joint counts in RA patients [34]. Astaxanthin has advanced to a randomised, placebo-controlled RA trial showing improvements in disease activity, function, and inflammatory markers [35], and hard-shelled mussel (*Mytilus coruscus*) lipid extract has shown benefit in a randomised controlled trial for RA [43]. The more extensive maturity of marine pharmacology is illustrated by the several marine-derived drugs already approved in other therapeutic areas (for example, the analgesic ziconotide and the anticancer agents trabectedin, eribulin and brentuximab vedotin), which demonstrates that marine chemical space can yield approved medicines and supports continued investment in marine anti-inflammatory leads [7]. By contrast, the great majority of compounds reviewed here, including the macroalgal fucans, marine microbial metabolites, sponge, coral and jellyfish products, remain at the preclinical stage and require validation in RA-specific in vivo models before clinical translation can be considered.

These clinical data should, however, be interpreted with caution (Table 2). The supporting trials are generally small (typically 38–76 participants), use heterogeneous and often non-standardised preparations and dosing, report a variety of symptomatic and biochemical endpoints rather than a consistent core outcome set, and are seldom compared against active RA therapy. Critically, none has demonstrated an effect on structural (radiographic) progression, the defining feature of a disease-modifying agent, and independent replication remains limited. The most advanced marine leads are therefore best regarded as symptomatic, adjunctive nutraceutical options that may complement conventional treatment in selected patients, rather than as emerging alternatives to established disease-modifying therapy.

## 6. Discussion

The compounds reviewed here demonstrate that marine organisms provide a chemically extensive arsenal of anti-inflammatory agents that converge on the core pathways driving RA. Macroalgal sulphated polysaccharides (from *Lobophora variegata* and *Lithothamnion muelleri*) reduce NO, TNF-α and leukocyte recruitment and attenuate joint inflammation in animal models [27,28]. Marine microalgae and heterotrophic protists supply the most clinically advanced leads, namely the omega-3 PUFAs EPA and DHA, prostaglandin-like oxylipins, and the carotenoids astaxanthin, fucoxanthin and β-carotene, which act through NF-κB, MAPK, JAK/STAT and Nrf2 signalling [29,34,35,36]. Marine bacteria and fungi, including deep-sea taxa, have contributed hundreds of recently described metabolites, such as the macrolactins and penicillinolide A, that inhibit iNOS, COX-2, and pro-inflammatory cytokines via NF-κB and TLR4 signalling [26,37,38]. Among the invertebrates, mussel lipid extracts (*Mytilus coruscus* and *Perna canaliculus*) provide the strongest human evidence [43,44,45], while sponges, corals (notably *Sinularia querciformis* and *Briareum excavatum*) and jellyfish offer mechanistically promising but earlier-stage leads [40,41,42,48,49,50,51,52,53,54,55].

A key question is what, beyond marine provenance, distinguishes these compounds from the many terrestrial natural products that also modulate NF-κB, MAPK, and oxidative stress pathways. Several features are potentially advantageous. Marine organisms occupy chemically extreme and highly competitive niches and elaborate structurally novel scaffolds, such as halogenated and sulphated metabolites, briaranes and amphilectane diterpenoids, which are rare or absent in terrestrial sources and may engage molecular targets more selectively [7]. Certain marine lipids and pigments, notably long-chain omega-3 PUFAs and the amphipathic ketocarotenoid astaxanthin, have favourable membrane partitioning and drug-like properties that support oral bioavailability and tissue distribution [29]. Finally, several of the most promising producers are microalgae, protists, and microorganisms that can be cultured or fermented, offering a scalable, reproducible, and sustainable supply and amenability to strain and metabolic engineering [26,29]. At present, however, these remain potential rather than demonstrated advantages, and direct evidence of therapeutic superiority over existing RA agents is lacking.

A consistent and clinically attractive feature of these compounds is their favourable safety profile: across the human and animal studies reviewed, marine lipid extracts and pigments produced little or no hepatotoxicity or other serious toxicity [35,43,44]. This contrasts with conventional RA therapies, where DMARDs, glucocorticoids and NSAIDs carry well-documented risks of cytopenias, hepatotoxicity, serious infection, osteoporosis, gastrointestinal bleeding and cardiovascular and renal toxicity [5,19,22,23]. Marine-derived compounds are therefore best positioned, in the near term, as well-tolerated adjuncts that may permit dose reduction in conventional agents, rather than as stand-alone replacements.

Several limitations temper these conclusions and define the research agenda. Much of the evidence derives from in vitro systems (typically LPS-stimulated macrophages) or from non-RA models of inflammation such as carrageenan- or monosodium urate-induced inflammation; because inflammatory mechanisms vary across conditions, extrapolation to RA must be cautious, and candidate compounds should be confirmed in validated RA models (collagen- or antigen-induced arthritis) before clinical claims are made [28,51]. For several extracts, the active constituents and exact molecular targets remain incompletely defined. The issues of compound stability, oral bioavailability, standardisation, and sustainable, ecologically responsible sourcing must be resolved. Here, cultured microalgae and fermentable heterotrophic protists and microorganisms offer a clear advantage over wild-harvested invertebrates by enabling scalable, reproducible, and traceable production [26,29]. Addressing these gaps through well-designed, RA-specific preclinical studies and adequately powered randomised trials is the essential next step. Despite several decades of mechanistic research and a growing catalogue of candidate molecules, the translation of marine-derived anti-inflammatory compounds into clinically meaningful RA therapies has so far been limited, and a realistic appraisal of their current therapeutic status is warranted.

## 7. Conclusions

Marine organisms are a rich and still under-exploited source of anti-inflammatory and immunomodulatory compounds with clear relevance to RA. The most compelling current evidence centres on marine microalgal and protist-derived omega-3 oils and carotenoids and on green-lipped and hard-shelled mussel lipid extracts, several of which have reached randomised controlled trials and are already available as nutraceuticals, while marine microorganisms represent a rapidly expanding frontier of novel chemistry. These compounds consistently target the NF-κB, MAPK, JAK/STAT, and Nrf2 pathways, which are central to RA, and do so with a favourable safety profile. Realising their therapeutic potential will require a deliberate shift from generic inflammation assays towards validated RA models and detailed clinical trials, alongside attention to standardisation, bioavailability and sustainable sourcing. With such evidence, marine-derived compounds could provide safer, complementary adjuncts to conventional RA therapy in selected patients, although, on current evidence, they are not substitutes for established disease-modifying treatment. Future research should prioritise the comparative evaluation of the most promising candidates for RA, including microalgal- and protist-derived omega-3 oils, astaxanthin, and mussel lipid extracts, in well-designed, adequately powered clinical studies that employ standardised preparations and consistently validated disease-activity endpoints. Complementary mechanistic investigations that define the active constituents and molecular targets of these compounds, coupled with structure-activity optimisation of lead scaffolds, are equally warranted. Such integrated efforts will be essential to translate these promising nutraceuticals into evidence-based therapeutics and, ultimately, to broaden and improve the management options available to patients with RA.

## Figures and Tables

**Table 1:** Translational status of representative marine-derived anti-inflammatory compounds relevant to rheumatoid arthritis.

Compound/Source	Source Group	Key Molecular Targets	Evidence and Development Stage	Refs.
*Perna canaliculus* lipid extract (Lyprinol, Seatone)	Mussel	PGE2, LTB4, pro-inflammatory cytokines	RCTs in RA/OA; marketed nutraceutical	[45,47]
Microalgal/protist omega-3 oils (EPA/DHA; *Schizochytrium*, *Nannochloropsis*)	Microalgae/protists	NF-κB; eicosanoid balance	RCT in RA (DHA); marketed supplement	[34]
Astaxanthin	Microalgae	NF-κB, MAPK, Nrf2, RANKL	RCT in RA; marketed supplement	[35]
*Mytilus coruscus* lipid extract (HMLE)	Mussel	TNF-α, IL-1β, PGE2 down; IL-10 up	RCT in RA	[43,44]
Fucoxanthin	Microalgae (diatoms)	NF-κB, MAPK, iNOS, COX-2	In vitro/animal; preclinical	[36]
*Lobophora variegata* heterofucans	Macroalga	NO, TNF-α, ROS	Animal arthritis; preclinical	[27]
*Lithothamnion muelleri* polysaccharides	Macroalga	CXCL1/CXCL2, leukocyte adhesion	Animal arthritis; preclinical	[28]
Macrolactins (e.g., 7,13-epoxyl-macrolactin A)	Marine/deep-sea bacteria	iNOS, IL-1β, IL-6, TLR4	In vitro; preclinical	[26,37]
Penicillinolide A	Marine fungus	NF-κB, iNOS, COX-2, cytokines	In vitro; preclinical	[38]
Excavatolide B; 11-epi-sinulariolide acetate (Ya-s11)	Corals	iNOS, COX-2; bone protection	Animal (incl. arthritis); preclinical	[48,49,50]
*Isostichopus badionotus* preparations	Sea cucumber	NF-κB, COX-2, TNF	Animal; preclinical	[39]
Sponge metabolites (*Hymeniacidon*; manoalide-type)	Sponges	COX/TXB2; PLA2; IL-6/IL-12	In vitro; preclinical	[40,41,42]
Jellyfish-derived (JF-AuNPs; Pelagia noctiluca)	Jellyfish	iNOS, NO	In vitro; preclinical	[53,54,55]

Abbreviations: COX-2, cyclooxygenase-2; DHA, docosahexaenoic acid; EPA, eicosapentaenoic acid; iNOS, inducible nitric oxide synthase; NO, nitric oxide; RCT, randomised controlled trial; TNF, tumour necrosis factor; PLA2, Phospholipase A2.; LTB4, LeukotrieneB4; TLR4, Toll-like Receptor 4.; MAPK, Mitogen-Activated Protein Kinase; CXCL1/CXCL2, Chemokine (C-X-C) Ligand 1 and Chemokine (C-X-C) Ligand 2; TXB2, Thromboxane B2.

**Table 2:** Summary of clinical studies of marine-derived compounds in rheumatoid arthritis and related arthritis.

Compound (Refs.)	Design	Participants (*n*; Population)	Comparator	Endpoints	Main Result
Green-lipped mussel (*Perna canaliculus*) lipid extract [45]	Double-blind, 3-mo parallel then 3-mo open	76; RA and OA	Mussel powder (no placebo)	Articular index, morning stiffness, grip, pain (VAS), function	Articular index, stiffness and function improved in both arms; pain/grip NS; modest effect
*Perna canaliculus* lipid extract (Lyprinol) [47]	Multicentre, 8-wk, open (uncontrolled)	60; hip/knee OA	None	VAS pain, Lequesne index	Symptomatic improvement (~53% at 4 wk, ~80% at 8 wk); no placebo control
Microalgal DHA (*Schizochytrium* sp.) [34]	Randomised, double-blind, placebo-controlled crossover	38; RA on stable therapy	Sunflower oil	Tender + swollen joint count, DAS28	Tender + swollen joints 13.9 -> 9.9 (*p* = 0.010); DAS28 4.3 -> 3.9 (*p* = 0.072)
Astaxanthin [35]	Randomised, triple-blind, placebo-controlled	60; RA	Placebo	DAS-28, HAQ, VAS, ESR, CRP, IL-6	DAS-28, HAQ, ESR, CRP reduced vs placebo (*p* < 0.05); IL-6 unchanged
Hard-shelled mussel (*Mytilus coruscus*) lipid extract [43]	Randomised, placebo-controlled, 6 mo	50; RA	Placebo	DAS28, CDAI, TNF-α, IL-1β, PGE2, IL-10	DAS28 and CDAI improved (*p* < 0.01); TNF-α, PGE2 down, IL-10 up

Abbreviations: CDAI, Clinical Disease Activity Index; CRP, C-reactive protein; DAS28/DAS-28, Disease Activity Score-28; DHA, docosahexaenoic acid; ESR, erythrocyte sedimentation rate; HAQ, Health Assessment Questionnaire; NS, not significant; OA, osteoarthritis; RA, rheumatoid arthritis; VAS, visual analogue scale.

## Data Availability

No new data were created or analyzed in this study. Data sharing is not applicable to this article.
